# Is inclusive education for children with special educational needs and disabilities an impossible dream?

**DOI:** 10.1111/bjep.12701

**Published:** 2024-06-18

**Authors:** Lisa Marks Woolfson

**Affiliations:** ^1^ Department of Psychological Sciences and Health University of Strathclyde Glasgow UK

**Keywords:** defining inclusive education, educational psychology in inclusive schools, practice framework, special needs research

## Abstract

**Background:**

Countries have been implementing inclusive educational practices for children with special educational needs and disabilities (SEND) for at least 30 years.

**Aims:**

Some issues continue to present as unresolved and will be examined in this paper with possible ways forward suggested. 1. There is still a lack of clarity around the definition of inclusion, its theoretical underpinnings, its implementation in practice and evaluation of success. 2. Teachers often still report the same problems of insufficient resources and express the same concerns about lack of skills and knowledge as reported in the early days. 3. A key question is, do children with SEND achieve better outcomes in inclusive educational settings?

**Discussion and Conclusions:**

The paper argues that an overarching executive framework applied to the education of children with SEND is needed to provide a common frame of reference that can be shared by educators, policymakers and researchers. New ways of resourcing inclusion are discussed including supporting collaboration between mainstream and special schools to better utilize the expertise located in special schools. The paper examines the evidence for improved academic and social outcomes for learners with SEND in inclusive schools and proposes that psychological outcomes now need to be measured too. It further suggests that future research needs to drill down to the level of teacher classroom instruction rather than rely on the broader mainstream school–special school comparison.

## INTRODUCTION

Countries have been implementing inclusive practices not least since the Salamanca statement ( UNESCO, 1994) and the Convention on the Rights of Persons with Disabilities (United Nations, 2006). Teachers in early research studies were broadly positive about the principle of inclusion but less satisfied about the training, physical and human resources provided to support it, with a variety of opinions about which populations of children with special needs it was really appropriate for (Cornoldi et al., 1998; Forlin et al., 1996). And tellingly after over 30 years of practical experience, many teachers in mainstream schools are still reporting broadly similar problems in implementing inclusive practices (de Boer et al., 2011; Nilholm, 2021; Woodcock & Marks Woolfson, 2019), and continue to feel they lack the necessary knowledge to create effective inclusive classrooms (e.g., Crispel & Kasperski, 2021; Göransson & Nilholm, 2014). Is inclusive education an impossible dream?

## PRINCIPLES

### Defining inclusive education

The inclusive education paradigm grew from concerns in the 1960s about the value of segregated education for children with special needs, and was influenced by the civil rights movement in United States and school improvement and school effectiveness developments in United Kingdom (Ainscow et al., 2006; Connor et al., 2008). From ‘integrating’ (some) children with special needs into mainstream schools, the concept grew to the further‐reaching goal of changing pedagogical practices and systemic structures to ensure that the school community accommodated not only diversity of ability but also differences of race, ethnicity, gender, family background, lifestyle and life experiences. While the internationally agreed principle of inclusive education now encompasses the broader concept of acknowledging diversity across the whole school population and aiming that all learners have a meaningful, quality educational experience (EASNIE, 2021; UNESCO, 2020), the focus in this paper is specifically on inclusion of children with special needs and disabilities (SEND).

This aspirational principle of inclusion and the concept of its being an ongoing process (UNESCO, 2017), a journey without a definite destination, is however difficult to operationalize for practical teaching and research purposes (Erten & Savage, 2012; Florian, 2014; Graham, 2020). Since the Salamanca statement, UNESCO publications have provided ever greater clarity as to the term's intended meaning regarding access to education and participation in the life of the school, as well as specific recommendations for policy and implementation (OECD, 2023; UNESCO, 2017, 2020). As a work in progress, there continues to be considerable variability in interpretation and implementation across different cultural and educational contexts. This can range from inclusion narrowly referring to placement of children with SEND in mainstream school in a separate class or separate unit, to the more visionary aspiration of creating a system that continually improves its policy and practice to remove barriers to ensure full student participation and engagement in the school community, both socially and academically (Erten & Savage, 2012; Forlin, 2015; Nilholm, 2021; Schwab, 2020). Inclusion is handled differently in different countries in terms of the proportion of children with SEND who attend mainstream schools. For example, although Australia and United States are committed to the same goals of recognition of the rights of children with disabilities, the number of segregated settings is increasing in Australia and decreasing in United States (de Bruin, 2019). A European survey carried out by Lebeer et al. (2011) reported a wide disparity between countries in the numbers of children identified as having SEND. Twenty‐five years on from the Salamanca statement, clear definitions of inclusion are still needed (Magnússon, 2019; UNESCO, 2020). Perhaps one explanation for teachers still feeling they lack skills and knowledge for inclusion is this confusing lack of consensus about what inclusion looks like in practical terms. Despite international agreements as to its aims and principles, it can range from placing a child with SEND to have their needs addressed within existing school structures, to challenging and changing those very structures, with a variety of approaches in between these two positions. Moreover, that classroom teachers have limited power to change a district‐wide or country‐wide system is now being recognized. For example, a key principle of a recent European report was that there should be a single legislation and policy framework underpinned by the concept of equity for all learners (EASNIE, 2021). The onus for successful implementation of inclusive education cannot only be on the class teacher.

### Theories and models that impact on inclusion of children with SEND

As well as an absence of an agreed definition of inclusion, it has been suggested that the area lacks theory to guide educational practice (Hornby, 2015; Nilholm, 2021). There are though several educational psychology theories that are usefully applied to inclusive education in the classroom. Some educators may be guided by a single theory to inform their practice while others may take a more eclectic approach applying several theories in their work, depending on the child's needs and curricular goals (Odom, 2016). Many teachers would see themselves as applying behaviourist theory in inclusive classrooms, for example to break down tasks into small manageable steps and to identify suitable reinforcers, both for learning and for management of behaviour in children with SEND. Cognitive theory informs how teachers can support retention of learning with mnemonics and development of metacognitive skills, and constructivist theory can inform a facilitative approach where teachers arrange structured learning experiences in inclusive environments to help learners construct their own understandings (Akpan & Beard, 2016; Al‐Shammari et al., 2019; Lenjani, 2016). Vygotsky's and Bruner's theories inform the social interactional role that effective teachers use, by structuring the task to enable learning and providing prompts and modelling next steps (Lourenço, 2012; Wood et al., 1976). The Theory of Planned Behaviour (Ajzen, 1991) has been applied in numerous research studies to examine teachers' intention to include learners with SEND, and the practice of inclusive behaviours (MacFarlane & Marks Woolfson, 2013; Urton et al., 2023; Wilson et al., 2016; Yan & Sin, 2015).

Beyond the level of the individual child in the classroom, systems theory provides a useful multi‐dimensional guide to practice by recognizing the range of levels, and social communities (Bronfenbrenner, 1979; Sameroff, 2010) at which inclusion and more pertinently exclusion of vulnerable groups of children can operate (Qvortrup & Qvortrup, 2018). A further aid to our understanding is the recognition that educational subsystems are socially constructed through interactions. Rapp and Corral‐Granados (2021) suggested that this perspective could usefully promote examination of how interactions and communications between school administrators, teachers, researchers and policymakers operate to include or indeed exclude particular groups of students.

The paradigm of the medical model has had a significant universal influence on the education of children with SEND by viewing educational intervention as a response only to the child's diagnosed disabilities, without taking into account individual, behavioural, social or contextual features that influence the child's functioning. This deficit‐focused model when applied to education has the undoubted benefit of resource allocation often following the diagnostic label. It can also lead to better teacher understanding of the nature of the child's identified difficulties. Such labelling though, concentrated on identification of difference and deficit, can be accompanied by negative outcomes such as experience of stigma, bullying and low self‐esteem (Algraigray & Boyle, 2017; Lauchlan & Boyle, 2007; Werner & Shulman, 2015). The rights, equity and social justice model guides practice by arguing for a child's right to non‐discriminatory education in their local mainstream school and accommodation of diversity without focusing on difference (e.g., Goldan et al., 2022; Norwich, 2014b). This latter model is linked to wider notions of an inclusive society. Indeed, recognition of the rights of children with disabilities was a key driver for initial steps to ensure children with special needs were not automatically placed in segregated special schooling as had been the policy prior to 1980s (Florian, 2014). Alongside this is an individual‐social model dichotomy, in which the former views disability as a problem of the individual, a personal tragedy, while the latter, influenced by the disability rights movement, views disability rather as a function of society's (poor) response to the individual's impairment (Gallagher et al., 2014; Oliver, 2013). The social model then is essentially political, with the goal of removing physical and social barriers experienced by disabled people in their daily life. It views disability as constructed by an uncaring and discriminatory society.

The concept of neurodiversity is an extension of the social model and may similarly be considered primarily political. It was initiated by people with autism and relates to the disability rights movement in extending the principle of an inclusive society to be one that respects and values neurological differences, and recognizes the strengths of neurodiverse individuals, in contrast to viewing difference as an abnormality or deficit to be fixed, feared or stigmatized (Krcek, 2013; Zaks, 2023). The term ‘neurodiversity’ has begun to be used in recent years by some practitioner educational psychologists as a generic term instead of specific learning difficulties, mostly but not only referring to autism, ADHD, DCD and dyslexia (Doyle, 2020). It is worth pointing out that the term, ‘neurodiversity’ was introduced by an Australian sociologist, Judy Singer, in her honours thesis, with the aim of offering a positive view of difference. Terms such as ‘neurodivergent’ and ‘neurotypical’ therefore are not underpinned by neurological differences or medical diagnoses, but are socio‐political constructs. Armstrong (2017) suggested that a neurodiversity‐based approach can provide us with a more nuanced conceptualization of disability that encourages educators to think about building on strengths. Rather than remedial work on deficits, teachers' efforts can be directed towards finding solutions and supportive strategies to help learners achieve access. Capability theory as applied to education similarly proposed moving away from assessing deficits and needs, to strengths and opportunities for achieving potential (Norwich, 2014a; Sen, 2014; Terzi, 2014; Walker, 2006).

Medical and social models of disability were integrated in The International Classification of Functioning, Disability and Health (ICF) which was developed by the World Health Organization as an interdisciplinary, holistic approach to the classification of the needs of people with disabilities (WHO, 2001). The capability approach and the ICF have several features in common. They are both holistic, looking beyond health conditions and limitations to consider contextual and personal influences on functioning (van der Veen et al., 2023). The ICF makes use of a biopsychosocial model. This model acknowledges there is an interplay between physical, social, environmental and psychological aspects in health and illness and advocates that a collaborative interdisciplinary intervention approach is needed, rather than only addressing biomedical features of illness (Bolton, 2022; Engel, 1977). A children's version, The International Classification of Functioning, Disability and Health—Children and Youth (ICF‐CY) was derived from the adult ICF to facilitate a holistic approach to assessment and intervention for children with SEND (Simeonsson, 2009; Simeonsson et al., 2003) Portugal and Switzerland were early adopters of ICF‐CY as a national mandatory tool for assessment in special education to inform intervention plans but it has not been widely used elsewhere (Hollenweger, 2011; Lebeer et al., 2011; Norwich, 2016; Sanches‐Ferreira et al., 2014).

It would seem then that inclusive education has several widely accepted psychosocial theories and models that are usefully being applied to aid understanding and improve inclusive classroom practices. Certainly there are not yet agreed ‘executive’ frameworks with a broad range of application within the system, but rather theoretical approaches and models that are applied in specific situations or to specific parts of the system (Kelly & Marks Woolfson, 2017), for example, applying behaviourist principles to managing child behaviour in the classroom. In this paper, ‘framework’ signifies a structured, overarching multilevel system subsuming concepts and theories within its overview. Its purpose is to provide a coherent system for translating theories and models into evidence‐based practice with a checklist of items for consideration. The terms ‘theory’, ‘model’ and ‘framework’ are often used interchangeably in research, policy and practice (Nilsen, 2020a), but the models that have been discussed here, medical, social, biopsychosocial seem more aligned to theory than to frameworks. Nilsen (2020b) suggested that models can be viewed as similar to theories but more descriptive and without the explanatory potential of a theory.

The biopsychosocial model has potential for contributing to a shared, overarching executive framework for inclusion of learners with SEND, yet its practical application, ICF‐CY, has not been used at systems level for educational assessment in United Kingdom and international take‐up generally for children with SEND has been limited. This is perhaps due to the time‐consuming nature of its inter‐professional information collection and also the ICF‐CY's medical diagnostic focus, which may not provide sufficient information to aid intervention planning for education professionals (Norwich, 2016).

## ARE OUTCOMES BETTER IN INCLUSIVE SETTINGS FOR CHILDREN WITH SEND?

Going beyond acknowledgment of the right of all children to be educated in their local mainstream schools, a key question is, do children with SEND achieve better outcomes in regular schools than in special schools? In order to decide if there are improved child outcomes in inclusive settings, it is necessary to be able to make comparisons between defined groups. However, researchers seeking to answer this question have experienced difficulty in finding studies that compare inclusive educational settings with self‐contained special classes or special schools that are specific in identifying the type and severity of disabilities of study participants, and indeed are sufficiently specific about what how their study defined ‘inclusive’ as discussed above. Reviewing papers on inclusive education published 2001–2005 Lindsay (2007) found only 14 out of 1373 studies provided a methodologically well‐designed comparison from which conclusions could be reliably drawn. Buchner et al. (2021) found in their study of seven European countries, that children with SEND in ‘inclusive’ educational settings might be learning independently in a mainstream class, or could have support from a teaching assistant, or a second support teacher in the classroom, or could even be in a special class within a mainstream school. Each of these quite different settings was described as inclusive and has quite different implications for what factors may contribute to any positive effects found. In their systematic review, Dell'Anna et al. (2020) reported that out of 1338 studies on children with moderate, severe and complex disabilities, only 18 were of sufficient methodological quality to infer effects of inclusive schooling.

### Academic outcomes

Studies report either cautiously positive effects of inclusive education or no difference between mainstream and special settings. Baker et al. (1994) concluded that SEND pupils in regular classes did better academically than those in non‐inclusive settings, with small but nevertheless worthwhile effects. Peetsma et al.'s (2001) Dutch longitudinal study compared matched pairs of primary‐aged pupils with mild learning and behaviour problems in mainstream and special schools. After 4 years, the mainstream group had made greater academic progress in language and mathematics than their matched pairs in special education. Ruijs and Peetsma's (2009) international review of findings on children with mild to moderate learning and behavioural disabilities concluded there were neutral to positive effects of inclusive education for academic achievement, although some studies were descriptive and without a special education control group. Dessemontet et al. (2012) reported slightly more progress in literacy skills for included children with learning disabilities in general mainstream classrooms, and no differences in progress in mathematics. de Graaf and de Graaf (2016) also found better academic outcomes for learners with Down syndrome in regular education settings. Dell'Anna et al.'s (2020) review identified similarly positive findings for children with moderate, severe and complex disabilities.

### Social outcomes

Academic outcomes though are only a narrow part of child's school experience. A driving force for many parents is that they anticipate increased social opportunities and resulting developmental benefits for their children with SEND through attending their local mainstream school. Children with SEND typically have reduced opportunities for social interaction and friendships outside the home and outside school, compared to their neurotypical peers (Taheri et al., 2016, 2017). Baker et al. (1994) reported a small positive effect on the social development of children with special needs and Dell'Anna et al. (2020) found a reduction in challenging behaviour. However, others have argued that studies of social inclusion or social status of SEND pupils show fewer positive findings than those on social development. Studies report marginalization of pupils with SEND, fewer interactions with non‐SEND peers and social isolation when not engaged in academic activities (Ballard & Dymond, 2016; Dell'Anna et al., 2020; Kemp & Carter, 2002; Ruijs & Peetsma, 2009). Children with SEND were less often nominated as someone's friend in sociometric studies and less often nominated as popular (Avramidis, 2013; Estell et al., 2008). If social inclusion is one of the aims of mainstreaming children with SEND, then inclusive education may not be succeeding in this very well. Often though studies compared children with SEND to peers without SEND in inclusive settings. If the comparison is children with SEND in mainstream versus children with SEND in special classes or schools, then findings on social outcomes are more positive (Lindsay, 2007; Peetsma et al., 2001). It may be that children with SEND in mainstream do not share the same social experiences and benefits of mainstream as their neurotypical peers but are benefiting there beyond their peers with SEND in special educational settings. This suggests that schools still have work to do to ensure optimal social inclusion for learners with SEND. They need to extend their efforts beyond formal academic learning opportunities for students with SEND, to facilitating their social participation. Well‐being and mental health outcomes too could usefully be evaluated as a measure of the effectiveness of inclusion, beyond academic and social outcomes. There is a role here for data to be gathered on a range of psychological outcomes for students with SEND (OECD, 2023).

## IT IS NOT MAINSTREAM OR SPECIAL SETTING: IT IS WHAT HAPPENS IN THE CLASSROOM

### Instructional practices

Lindsay's (2007) review concluded that it was necessary to examine mediators and moderators to establish an evidence‐based rather than an equity, rights‐based approach to optimizing educational outcomes for children with SEND. Labelling classroom environments as special or inclusive for research purposes is problematic for interpretation of findings as the lack of definition of these terms means that we do not know how comparison classrooms differed in their approach to teaching and learning. Positive outcomes may not be a function of whether it was a mainstream or special school setting, but instead linked to instructional practices, for example, learner‐centred teaching, supportive climate, expectations of performance. Klang et al.'s (2020) Swedish study was designed to examine this and found similar amounts of teacher‐centred and learner‐centred time for each group. Earlier though, Matzen et al.'s (2009) observational study, comparing the experiences of three learners with SEND in both special and regular classes, reported more academic opportunities for them, 85%–96% in inclusive settings and only 48%–56% in the self‐contained special classroom. Klang et al. (2020) also found higher expectations of SEND pupils in mainstream settings but better support for social participation in special education settings.

### Teacher attitudes

For successful implementation of inclusive education policies, educators tasked with implementing them in practice in the classroom need to be positive about them, and need to want to make inclusion of learners with SEND work (Avramidis & Norwich, 2002; van Mieghem et al., 2020; Wilson et al., 2016). As a result of this assumption, there has been considerable research on teacher attitudes to inclusive education. Some studies report positive teacher attitudes (Avramidis et al., 2000; Guillemot et al., 2022; Wilson et al., 2019), while others have found evidence of neutral or negative attitudes (Avramidis et al., 2019; de Boer et al., 2011; Thaver & Lim, 2014). Country‐specific factors such as socioeconomic status and societal perception of disability influence these attitudes, as well as a complex interplay between teacher demographic factors (Guillemot et al., 2022; van Steen & Wilson, 2020). Teacher self‐efficacy for delivering effective inclusive classrooms is also recognized as a factor that contributes to attitudes (Brady & Marks Woolfson, 2008; Park et al., 2016; Schwab et al., 2017; Wray et al., 2022; Yada et al., 2022). Training teachers on how to implement instructional practices in inclusive classrooms has been demonstrated as necessary and effective in changing attitudes and increasing knowledge and self‐efficacy in both preservice education and continued professional development of teachers in post (Kurniawati et al., 2016; Sharma & Nuttal, 2016; Wilson et al., 2020; Woodcock & Vialle, 2016).

## RESOURCING

Perhaps though, another important reason for any teacher reticence around inclusive practices is that too much is being asked of them without adequate resourcing. The term ‘resourcing’ can cover time allocation for planning and developing curricula, availability of instructional materials, specialist and non‐specialist staff, professional development activities, each of which has budgetary implications. The Salamanca Statement made it clear 30 years ago that implementation of its priorities had expenditure implications that required to be addressed. How best to achieve the effective use of resources to promote inclusion has continued to be a live issue (UNESCO, 2017). Not surprisingly then, resourcing has emerged as an important factor in teachers' attitudes to inclusive education and also in the retention of teachers in SEND roles (Avramidis & Norwich, 2002; Billingsley & Bettini, 2019; Gilmour & Wehby, 2020; Goldan & Schwab, 2020; Guillemot et al., 2022; Woodcock & Marks Woolfson, 2019). For example, it is likely that budgetary resource constraints together with an emphasis on academic curriculum targets, resulted in proportionally more school suspensions of learners with SEND in England than those without SEND, especially where there was challenging behaviour (DfE, 2024). Practitioner educational psychologists play a valuable role in assessing special educational needs, supporting learners with SEND and promoting inclusive practices in schools, but the majority of English local authorities have had difficulty recruiting and retaining sufficient educational psychologists to even carry out the statutory assessments for education and health care (EHC) plans (DfE, 2023), let alone any further input. Not all children with SEND require EHC plans but those who require additional support need them. In its *Trends Report 2024*, the American Psychological Association reported a similar shortage of school psychologists in United States who are needed for individualized education programmes, the equivalent legal document for children with SEND. The alarming picture here is one of teachers struggling to accommodate a diverse range of academic and behavioural needs in their classrooms within existing resource allocations and without sufficient support from the wider regional education system, alongside a bottleneck of children with SEND waiting to be assessed as to which services they require.

How then is funding allocated and to what extent does this promote inclusive educational practices? Meijer and Watkins (2019) identified two main funding models:

1. *Input model* that allocates funding based on measurement of special educational need and the number of learners with that need in a school or district. Meijer and Watkins (2019) point out that a negative effect of this is that the more learners with SEND are identified, the more funds are then allocated. The effect of this is that low achievement draws funding. Budgetary support is linked to labelling and diagnosis of difference, and therefore works against the guiding principles of inclusive education.
2. *Throughput model* is based not on need, nor on input or indeed output, but on special educational support services that the school or district must provide. A negative consequence of the throughput model is that as funding is not dependent upon outcomes, effective inclusive educational practice is not incentivised.

Additionally, an *output model* is based on performance outcomes, although this model tends not to be found in practice alone but rather combined with a throughput model (Goldan, 2021). The issue is how to allocate funding in a way that is not only sufficient for purpose and fairly distributed according to need, but in a way that also fosters effective inclusive educational practices. This continues to be a challenge for many European countries who still allocate SEND resourcing much as they did in the 1990s (Meijer & Watkins, 2019), even in today's very different social context with its transformative expectations around equity and neurodiversity. A recent OECD report addressed this issue by recommending that education systems should not use only targeted resource funding for students needing additional support but should also use their main allocation budgets (OECD, 2023).

## LOOKING TO THE FUTURE

This paper has examined a range of factors that influence the effectiveness and sustainability of inclusive education. What are possible ways forward to move a visionary educational aspiration to a sustainable reality for the future?

1. There is still work to be done by educators, administrators and researchers towards reaching a shared definition of inclusion and global consensus understanding of what this means for the participation of learners with SEND in their local community schools. This would better allow interpretation and understanding of international data and facilitate designing research, comparing and sharing findings, and help identify best practice.
2. Attention should be turned to developing a shared executive theory‐based and evidence‐based framework to inform inclusive practices both in the classroom and within the wider district education system. This framework needs to be able to offer a common language to healthcare, social care and education professionals as well as academic researchers. The biopsychosocial model is a good candidate for underpinning such an overarching executive framework within which approaches with a narrower range of convenience can be located.
3. Resourcing models need to be redesigned to promote inclusive practices. There is still a place for bottom‐up resourcing, in response to assessment of individual special needs. However, alongside this, resource models need a significant top‐down element so that the main funding allocation can facilitate reimagining education systems and developing innovative district‐ and country‐wide support services that promote inclusion. Funding resource should support collaboration between mainstream and special schools to better utilize the expertise located in special schools. Currently, there is no resource to encourage sharing such skills and knowledge, so only small‐scale initiatives take place. In addition, funding should reflect successful outcomes and provide additional resources where needed. A combination of funding models may be the way forward for resourcing.
4. There is a key role for educational psychology research. Research on the success of inclusive education needs now to go beyond measurement of academic and social outcomes to investigate social–interactional factors that operate in successful inclusive classrooms and school communities. This could include, for example, identifying effective instructional practices, the use of learner‐centred teaching, or how to create a positive social climate for learners with SEND that facilitates increased social participation. Research is also needed on psychological outcomes for students with SEND, such as mental health and well‐being, anxiety, stress, life satisfaction.
5. Improved preservice training of teachers is needed to better prepare new teachers for inclusive classrooms. This should include practical experience of teaching learners with SEND as a central element of the training programme to allow new teachers to build relevant assessment, knowledge and instructional skills for addressing neurodiversity in the classroom. Coaching and mentoring by teachers experienced in inclusive education could be provided to support teachers starting out.

## CONCLUSIONS

The first two recommendations above are each about finding commonalities in language and implementation frameworks. This involves international collaboration between countries, and professional collaboration between educational psychologists, educators, health and social care practitioners and administrators within countries. More consensus on these issues is perfectly possible by continuing to advance this specific agenda through appropriate international avenues such as UNESCO and EASNIE conferences, and national interdisciplinary symposia. The recommendations about new ways of resourcing inclusive education and improved preservice teacher training have in common that they both require a major conceptual shift so that inclusive education of learners with SEND is viewed holistically as a central part of the education agenda, rather than as additional to the main task of funding/educating learners without SEND. Redesigned funding is an important recommendation as, even with new thinking and good intentions from education professionals on the ground, without reassessing funding models, there is a limit to the potential for moving inclusive education forward.

Educational psychology researchers and practitioners have a role in advancing the above recommendations. Effective inclusive education for children with SEND is indeed possible and we are working our way ever closer to that goal.

## AUTHOR CONTRIBUTIONS

**Lisa Marks Woolfson:** Conceptualization; writing – original draft; visualization; writing – review and editing.

## CONFLICT OF INTEREST STATEMENT

No conflict of interest.

## Data Availability

Data sharing not applicable to this article as no datasets were generated or analysed during the current study.
